# Saliva Immunoglobulin Concentrations Are Associated with Colostrum Intake and with Serum Concentrations in Newborn Calves

**DOI:** 10.3390/ani15152224

**Published:** 2025-07-28

**Authors:** Flávio G. Silva, Elsa Lamy, Paulo Infante, Cristina Conceição, Joaquim L. Cerqueira, Joana M. Ramalho, Marta González-Cabrera, Pedro Caetano, Luís Martins, Severiano R. Silva, Alfredo Pereira, Lorenzo E. Hernández-Castellano

**Affiliations:** 1 Veterinary and Animal Research Centre & Al4AnimalS, Department of Animal Science, University of Trás-os-Montes e Alto Douro, Quinta de Prados, 5000-801 Vila Real, Portugal; fsilva@uevora.pt (F.G.S.); cerqueira@esa.ipvc.pt (J.L.C.); ssilva@utad.pt (S.R.S.); 2 MED-Mediterranean Institute for Agriculture, Environment and Development, and CHANGE—Global Change and Sustainability Institute, University of Évora, Pólo da Mitra, Ap. 94, 7006-554 Évora, Portugal; cristinaconceicao@uevora.pt (C.C.); pcaetano@uevora.pt (P.C.); lmlm@uevora.pt (L.M.); apereira@uevora.pt (A.P.); 3 Center for Research and Development in Agrifood Systems and Sustainability, Polytechnic Institute of Viana do Castelo—Agrarian School of Ponte de Lima, Rua D. Mendo Afonso, 147, Viana do Castelo, 4990-706 Ponte de Lima, Portugal; 4 CIMA—Centro de Investigação em Matemática e Aplicações, Universidade de Évora, 7006-554 Évora, Portugal; pinfante@uevora.pt; 5 Department of Veterinary Medicine, School of Sciences and Technology, University of Évora, Pólo da Mitra, Ap. 94, 7006-554 Évora, Portugal; joana.ramalho94@gmail.com; 6 IUSA-ONEHEALTH 4, Animal Production and Biotechnology Group, Institute of Animal Health and Food Safety, Universidad de Las Palmas de Gran Canaria, Campus Montaña Cardones, s/n, 35413 Arucas, Spain; marta.gonzalezcabrera@ulpgc.es

**Keywords:** dairy, IgA, IgG, total protein, transfer of passive immunity, saliva, colostrum

## Abstract

The transfer of passive immunity (TPI) is vital for newborn calf health and is typically assessed through blood sampling to measure serum or plasma IgG or total protein levels. Saliva offers a less invasive alternative. This study evaluated the potential of saliva for assessing TPI. Saliva and serum samples were collected from 20 calves at birth and on days 1, 2, and 7 of life to measure IgG, IgA, IgM, and total protein concentrations. Colostrum fed to the calves was also analyzed for gross composition and immunoglobulin content. Colostrum intake and composition were associated with saliva IgG (R^2^ = 0.48; *p* = 0.019), saliva IgM (R^2^ = 0.73; *p* = 0.02), and saliva total protein (R^2^ = 0.41; *p* = 0.043). Serum IgG and IgA levels were predicted by saliva IgG (*p* < 0.001; R^2^ = 0.33) and IgA (*p* < 0.001; R^2^ = 0.13), respectively, though predictive accuracy varied by calf age (IgG: *p* < 0.001; R^2^ = 0.40; IgA: *p* < 0.001; R^2^ = 0.91). In conclusion, the results suggest that both saliva IgG and IgA concentrations could be used to predict IgG and IgA serum concentrations. Nevertheless, further studies are still needed to clarify the best time for sample collection.

## Simple Summary

Colostrum intake is essential for the transfer of passive immunity from the dam to the newborn calf. The assessment of the transfer of passive immunity is often performed by measuring blood antibody concentrations. This study explored the potential of saliva as a non-invasive and more accessible alternative to assess the transfer of passive immunity. Thus, the relationship among saliva antibody concentrations, colostrum intake, and serum antibody levels was determined. The results showed a significant association between saliva and serum antibody concentrations, highlighting the potential of saliva as a viable, non-invasive method for evaluating calf immune status.

## 1. Introduction

The transfer of passive immunity (TPI) from colostrum is essential to ensure the health and welfare of calves [1,2,3]. Successful TPI can be determined by measuring either serum immunoglobulin (Ig)G or serum total protein (TP) concentration within 1 to 7 days (ds) of age and can be classified as excellent (≥25.0), good (18.0–24.9), fair (10.0–17.9), and poor (<10 g/L of IgG; [4]). Although the relevance of the assessment of TPI is increasing among dairy farmers, it is still not a common procedure [5,6]. One possible reason is that the assessment involves blood sampling. This invasive procedure requires specialized training and, depending on national legislation, the presence of a veterinarian. Therefore, more feasible and less invasive methods to assess TPI on dairy farms are required.

Saliva is a watery fluid that contains proteins, ions, and other organic compounds synthesized mainly by the salivary glands, with some compounds derived from blood [7,8]. The concentration of maternal Igs (i.e., IgG, IgA, and IgM) increases in the calf’s blood following colostrum intake (C.INT) [2,9]. These Igs help protect the calf against pathogenic microorganisms during the first weeks of life [10]. Among them, IgG has a relatively long half-life of approximately 28.5 days [11], whereas IgA and IgM decline more rapidly, with reported half-lives of 2.8 and 4.8 days, respectively [12]. Indeed, IgG, particularly the subclass IgG_1_, is the main Ig involved in the protection of the newborn calf [2,9] and has a molecular weight of approximately 150 kDa [13]. Immunoglobulin A, typically present in colostrum and milk as secretory IgA (410–434 kDa), plays a key role in protecting mucosal membranes [14]. Immunoglobulin M, with a higher molecular weight (900 kDa), is particularly effective in agglutination and complement fixation, contributing mainly by preventing septicemia [2,9]. The main advantage of saliva compared to blood is that its collection is easier and less invasive. However, the immunological components present in the saliva of newborn ruminants are not well described yet. Johnsen et al. [15] described a positive correlation between saliva and blood IgG, although some saliva IgG concentrations were below the detection limit of the analytical method. Recently, Berteselli et al. [16] described increased saliva IgA concentrations in newborn calves on day 2 of life, but they found no correlation between serum and saliva of either IgA or IgG. Information on other Igs in saliva after C.INT, such as IgM, has not been described yet. In addition, there is no direct information about the relation between the concentration of different Igs (i.e., IgG, IgA, and IgM) in bovine colostrum with those in newborn calf saliva.

Therefore, this study hypothesizes that IgG, IgA, and IgM concentrations in newborn calf saliva depend on C.INT and that these concentrations are associated with those found in blood serum. The aim of this study was (1) to evaluate the association between saliva IgG, IgA, IgM, and TP with C.INT and composition and (2) to evaluate whether saliva IgG and IgA concentrations could be used as predictors of serum IgG and IgA concentrations during the first week of life.

## 2. Materials and Methods

Detailed descriptions of animal management, sampling, and laboratory analysis were reported in a previous publication [17]. Briefly, 20 newborn dairy calves from a commercial dairy farm located in Évora (Portugal) were included in this study. Each animal was bottle-fed colostrum within the first 3 h after birth, and C.INT was recorded. Colostrum was provided from the farm’s colostrum bank, consisting of previously frozen colostrum from individual cows. From days 1 to 3, the calves received 3 L of transition milk twice daily, followed by milk replacer (Bovimilk, Vetlima, Vila Nova da Rainha, Portugal) until day 7. Fresh water was available ad libitum. Composite colostrum samples were collected from each bottle before consumption, and saliva and serum samples were collected on d 0 (right after birth and 30 min before C.INT) and then on days 1 (24 h), 2 (48 h), and 7 (168 h) after birth. During these days (i.e., 1, 2, and 7), saliva samples were collected within 2 to 7 h after milk intake to avoid milk residuals in the oral cavity. Saliva was collected using Salivette cotton swabs (Sarstedt GmbH, Nümbrecht, Germany). Colostrum gross chemical composition (i.e., dry matter (DM), protein, and fat) was analyzed by the AOAC Official Methods, and colostrum somatic cell count (SCC) was determined using a DeLaval cell counter (DeLaval, Tumba, Sweden). Total protein in serum and saliva was analyzed by refractometry (Zuzi, model 50303020, Auxilab, Navarra, Spain; range of 0–12 g/dL and resolution of 0.2 g/dL) and by the Bradford method [18], respectively. Commercial ELISA kits (Bethyl Laboratories, Montgomery, TX, USA) were used to determine Ig (i.e., IgG, IgA, and IgM) concentrations in colostrum, saliva, and serum [16].

Statistical analyses were performed using R, version 4.4.1. [19]. A power analysis was performed using the pwr package to determine the minimum number of animals required to detect a significant association at α = 0.05 with 90% power. An expected correlation of 0.7 was used [15] to calculate the sample size. A linear mixed effects model (LMM) with time (d 0, 1, 2, and 7) as a fixed effect and the calf as a random factor was used. The Fisher’s LSD test was used to compare statistical differences between different levels of time when no violations of the model’s assumption occurred. A Bonferroni correction was applied to pairwise comparisons of models with unequal variances to reduce the likelihood of type I errors. To assess the association between colostrum composition, C.INT, and saliva and serum variables, a backwards stepwise multiple regression model was used. The model included colostrum composition variables (i.e., DM, protein, fat, SCC, IgG, IgA, and IgM), C.INT, and calf age at first C.INT (i.e., time in hours between birth and C.INT) as explanatory variables. Saliva and serum Ig (i.e., IgG, IgA, and IgM) and TP concentrations on day 1 were included as outcome variables. Residuals of each model were visually inspected for normality and homoscedasticity. No transformation was required for the response variables in this analysis. Multicollinearity among explanatory variables was assessed using the Variance Inflation Factor.

To analyze the predictive effect of saliva Igs on serum Ig concentrations, an initial exploratory analysis was performed using Spearman correlations between saliva and serum Ig, since the residuals of some variables did not follow a normal distribution. Linear and nonlinear models were fitted to explore the relationship between IgG and IgA concentrations in saliva and serum across four distinct time points (i.e., days 0, 1, 2, and 7). Each calf was treated as a unique experimental unit, allowing for the modeling of within-subject variability as a random effect. The initial model fitted was an LMM, including saliva IgG or IgA concentrations as a fixed predictor and time (i.e., days 0, 1, 2, and 7) as a covariate. An interaction term between these variables was included to account for potential time-dependent effects. A random intercept for each calf was specified to capture inter-subject variability:

$$\begin{array}{cc} {Serum}_{ij} & =\beta_{0}+\beta_{1}{Saliva}_{ij}+\beta_{2}{Time}_{j}+\beta_{3}{({Saliva}_{ij}*Time}_{j})+u_{i}+\varepsilon_{ij},\\ i & =1,\ldots 20,j=0,1,2,7, \end{array}$$

${Serum}_{ij}$ is the serum IgG or IgA concentrations for calf *i* at time *j*;

${Saliva}_{ij}$ is the salivary IgG or IgA concentrations for calf *i* at time *j*;

${Time}_{j}$ represents days after birth;

$\beta_{0},\beta_{1},\beta_{2},{and\beta }_{3}$ are fixed coefficients;

$u_{i}\sim N(0,\sigma_{u}^{2})$ is the random effect associated with calf i;

$\varepsilon_{ij}\sim N(0,\sigma_{\varepsilon }^{2})$ is the residual error term.

To identify the most appropriate models, several alternative structures were considered and compared using the Akaike Information Criterion (AIC) and likelihood ratio tests. These alternatives included (a) exclusion of the interaction between saliva and time; (b) simplification of the random effects structure; (c) nonlinear and additive mixed models to capture potential nonlinearity; and (d) incorporation of autocorrelation structures.

The final models were thoroughly evaluated to ensure compliance with statistical assumptions, including the normality and homoscedasticity of residuals. The structure of the random effects was also assessed. Significant values were considered as *p* < 0.05 and tendencies as *p* < 0.1.

## 3. Results

The minimum number of animals obtained from the power analysis was 17; however, 3 additional calves were included in the experiment to compensate for possible losses unrelated to the experiment.

**Figure 1:**
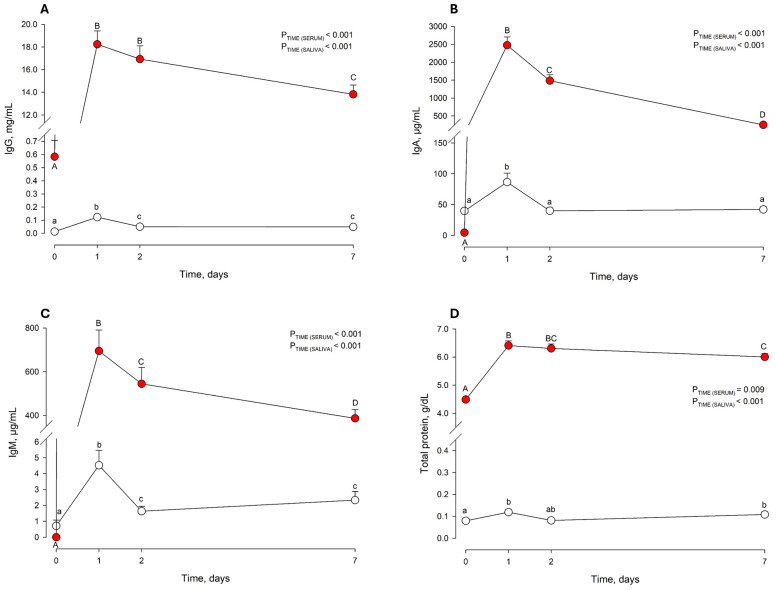
Saliva (○) and serum (●) immunoglobulin G (**A**), immunoglobulin A (**B**), immunoglobulin M (**C**), and total protein (**D**) concentrations across the experimental period (i.e., days 0, 1, 2, and 7). Different letters (a–c) indicate significant differences between days (days 0, 1, 2, and 7) in saliva. Different letters (A–D) indicate significant differences between days (days 0, 1, 2, and 7) in blood serum. Results are expressed as LSM ± SEM.

On average, 3.8 ± 0.64 L (mean ± standard deviation) colostrum was administered to each calf within 96 ± 73 min of birth. Descriptive statistics for colostrum composition are shown in Table 1. Colostrum samples with IgG concentrations ≥ 50 mg/mL and SCC < 400,000 cells/mL represented 35% and 15% of the total samples, respectively. Saliva and serum IgG, IgA, IgM, and TP concentrations changed over time, increasing after C.INT (i.e., d 1) and subsequently decreasing until day 7 (*p* ≤ 0.009; Figure 1).

### 3.1. Associations Between Colostrum Composition and Colostrum Intake and Saliva and Serum Immunoglobulins and Total Protein Concentrations

Stepwise multiple regression models were used to evaluate the associations between C.INT and colostrum composition and saliva and serum Ig and TP concentrations on day 1. The final models and corresponding coefficients are presented in Table 2.

Saliva IgG concentration was positively associated with C.INT and SCC (R^2^ = 0.48; *p* = 0.019). Saliva IgM concentration was positively associated with C.INT, SCC, and colostrum protein and IgA concentration and negatively associated with colostrum IgG concentration (R^2^ = 0.73, *p* = 0.02). Saliva IgA concentration was not associated with any colostrum variable (*p* = 0.165). Saliva TP concentration was positively associated with C.INT and colostrum IgM concentration (R^2^ = 0.41, *p* = 0.043). Serum IgG concentration was positively associated with C.INT and colostrum protein (R^2^ = 0.49, *p* = 0.018). Serum IgA concentration was positively associated with C.INT and colostrum protein and IgA concentration and negatively associated with colostrum IgM and IgG concentrations and with the time between birth and C.INT (R^2^ = 0.91, *p* < 0.001). Serum IgM concentration was positively associated with colostrum IgM and IgA concentrations, SCC, and with time between birth and C.INT (R^2^ = 0.66, *p* = 0.02). Serum TP concentration was positively associated with C.INT and colostrum protein and negatively associated with colostrum IgM concentration (R^2^ = 0.89, *p* < 0.001). Neither colostrum DM nor fat was included in any of the final models.

### 3.2. Predictive Models for Serum IgG and IgA Concentrations

To assess the predictive potential of saliva IgG and IgA concentrations for their respective serum concentrations across time (days 0, 1, 2, and 7), linear and nonlinear models were fitted.

#### 3.2.1. Spearman Correlations Between Saliva and Serum Immunoglobulins

Saliva and serum IgG concentrations were correlated on d 0 (ρ = 0.43, *p* = 0.058). Similarly, saliva and serum IgA (ρ = 0.40, *p* = 0.074) and IgM (ρ = 0.42, *p* = 0.057) concentrations were correlated on d 2. Saliva IgG and saliva IgA were correlated on d 0 (ρ = 0.54, *p* = 0.017), d 1 (ρ = 0.74, *p* < 0.001), and d 2 (ρ = 0.48, *p* = 0.033). Saliva IgG and saliva IgM were also correlated on d 0 (ρ = 0.62, *p* < 0.004), d 1 (ρ = 0.76, *p* < 0.001), d 2 (ρ = 0.44, *p* = 0.053), and d 7 (ρ = 0.73, *p* < 0.001). Saliva IgA and saliva IgM were correlated on d 1 (ρ = 0.73, *p* < 0.001) and d 2 (ρ = 0.65, *p* = 0.001). Serum IgG and serum IgA were correlated on d 1 (ρ = 0.62, *p* = 0.003), d 2 (ρ = 0.46, *p* = 0.036), and d 7 (ρ = 0.52, *p* = 0.017). Serum IgM was not correlated with either serum IgG or IgA. No other statistically significant differences (*p* < 0.05) or trends (*p* < 0.10) were observed.

#### 3.2.2. Predictive Models for Serum IgG

Two models were compared, namely, (i) a mixed-effects model including saliva IgG, time (as a continuous covariate), and their interaction (*p* < 0.001; R^2^ = 0.40) and (ii) a nonlinear fixed-effect model without time, where the relationship followed a quadratic trend (*p* < 0.001; R^2^ = 0.33). Although both models showed saliva IgG as a significant predictor (*p* < 0.001), the inclusion of time improved the model fit. The results are detailed in Table 3.

#### 3.2.3. Predictive Models for Serum IgA

The final model predicting serum IgA concentrations from saliva IgA concentrations was a model without random effects, with time as a categorical fixed effect and the response variable transformed into a power raised to 0.25 to satisfy normality and homoscedasticity (*p* < 0.001, R^2^ = 0.91; Table 4). After fitting the same model without accounting for the effect of time, saliva IgA concentrations remained a significant predictor of serum IgA concentrations (*p* < 0.001, R^2^ = 0.13; Table 4).

## 4. Discussion

This study is the first to evaluate the relationship between saliva and serum IgG and IgA in newborn calves while explicitly considering the effect of time after birth in statistical models. As previously described, saliva and serum IgG are positively correlated in newborn calves after C.INT [15]. Therefore, assessing the TPI through saliva could provide a feasible tool to evaluate colostrum management on dairy farms. The results support the potential of saliva as a non-invasive matrix to estimate TPI, as saliva Ig concentrations were positively associated with C.INT and, in the case of IgG and IgA, with their respective serum concentrations.

Although the relationship between serum Ig concentrations and C.INT and colostrum composition is well described [20,21,22], limited literature is available about the relationship between colostrum and saliva Ig and TP concentrations. As previously reported, colostrum and saliva Ig share the same antigenic properties [23], evidencing the interconnection of the mucosal immune system [24]. However, whether colostrum Ig can be found in the mucosal secretions of offspring remains unknown. As suggested by Smith et al. [25], colostrum IgG can reach nasal and lachrymal secretions of newborn lambs, whereas IgA and IgM have not been detected in these biological fluids. The positive associations found between C.INT and saliva IgG, IgA, IgM, and TP, as well as between colostrum IgA and saliva IgA, suggest that either these Igs can reach calf mucosal secretions or that Ig secretion at mucosal sites is increased by C.INT. A positive association between C.INT and serum IgG concentration has been described as either a linear [26] or a nonlinear relationship [27]. Nevertheless, the slope of this relationship declines with increasing age at first C.INT [22,28]. As most studies focus on IgG, the dynamics of IgA and IgM in newborn calves remain less explored. Unlike IgG and IgA, serum IgM concentrations were shown to be negatively related to the total mass of IgM intake [29]. As described by Puppel et al. [30], high-SCC colostrum (≥400,000 cells/mL) contains a lower IgG concentration than low-SCC colostrum (<400,000 cells/mL). However, maternal leukocytes have an important biological role in the newborn calf [3,31]. Therefore, colostrum SCC should be assessed together with colostrum microbiological load [32]. The positive associations found between saliva Ig and colostrum SCC suggest that maternal cells in colostrum may be important for the transfer of Ig from serum to saliva. However, high SCC can be indicative of infection, affecting the tight junctions and therefore increasing the transfer of cells from blood to mammary secretions [33,34]. In such a case, other immune components, such as Ig, are also expected to be increased in colostrum [35] and, consequently, in the serum of the offspring [36], although some authors have not reported enhanced absorption of immune components in newborn calves [37] and goat kids [38]. Nevertheless, in the present study, a positive association with SCC and Ig in serum was only found for IgM. In newborn ungulates, Ig absorption occurs by a non-specific endocytic process in the proximal part of the small intestine [39]. Therefore, this absorption process depends on the intestinal permeability and on the binding surface of the enterocyte membrane, which can be saturated by other macromolecules [38]. In addition, Igs (i.e., IgG, IgA, and IgM) are present in different concentrations in colostrum, with IgG accounting for approximately 86% of the total Igs [40], which are absorbed at different rates in the calf intestine [41]. This could explain the observed negative coefficients of colostrum IgG concentrations with saliva IgM and serum IgA concentrations. In addition, Igs constitute a large proportion of the TP content in colostrum [42], which helps explain the associations observed between colostrum protein and serum Ig concentrations, in agreement with the results reported by Thu Hang et al. [43]. Nevertheless, other colostrum proteins, like lactoferrin and lactoperoxidase, that contribute to gut protection against pathogens may also improve the efficiency of Ig absorption [3].

Few studies have evaluated the potential of saliva for assessing TPI in newborn dairy calves [15]; however, the correlation between serum and saliva IgG likely depends on circulating IgG concentration. In fact, Berteselli et al. [16] showed increased saliva and serum Igs after C.INT, although no correlation between Igs in both fluids was detected. These authors also found that saliva IgG and IgA were correlated after C.INT, which agrees with the positive correlation observed in the present study between IgG and IgA in both saliva and serum on d 1 and d 2. The present results highlight a predictive effect of saliva IgG on serum IgG concentrations, which decreases over time (Table 3). The relationship between saliva and serum IgG concentrations was evident and remained significant even after the exclusion of time in the model, indicating a stable association between saliva and serum IgG concentrations throughout the first week of life. The relationship between saliva and serum IgG, excluding the effect of time, exhibited a quadratic pattern, which suggests a physiological limit of IgG transfer from serum to saliva. This limitation is likely due to saturation of the transport mechanisms, similar to those described at the intestinal epithelium [27,44]. In contrast, the relationship between saliva and serum IgA concentrations was time-dependent. The inclusion of time as a categorical variable in the model improved the predictive capacity of saliva IgA compared to a model without time. This finding highlights that the dynamics of IgA during the first week are not linear and likely reflect rapid physiological changes in the mucosal immune system, such as increased epithelial permeability shortly after birth and maturation of salivary glands [45,46]. Therefore, the strong influence of time on IgA prediction supports the hypothesis that IgA transfer or secretion to saliva is not constant but evolves rapidly after C.INT and during early development. One hypothesis for the differences obtained between IgG and IgA could be related to the higher molecular weight and dimeric structure of colostrum-derived IgA [14]. In addition, IgG can even be transferred from blood to saliva through gingival crevices [47]. As the predictive model for IgA was higher than the model for IgG, future research, as suggested by Berteselli et al. [16], should consider saliva IgA concentrations as a candidate to assess TPI in newborn calves. However, the best sampling time for saliva IgA monitoring needs to be further investigated.

While this study could not differentiate between serum-derived and locally synthesized salivary IgA, it is important to note that active synthesis of saliva IgA likely starts later in life [48]. The potential presence of residual colostrum or transition milk Ig in the mouth of the calf while sampling is a possible confounding factor. In this study, residual Ig was avoided by collecting saliva samples before transition milk feeding or at least 2 h after the last meal. However, completely avoiding residual Ig is not possible and should be considered as a limitation of saliva sampling for TPI assessment. Therefore, predictive models built with saliva from newborn calves must be tested under this limitation to ensure their practical applicability in farm settings. Additionally, time of birth, and not of C.INT, was considered as the basis for subsequent sampling; thus, it is not possible to assess whether these changes in the transfer rate of colostrum Ig to serum and consequently to saliva are caused by the maturation of the intestinal epithelial cells, by a direct effect of C.INT, or by both [49]. Although ELISA cannot be used on the farm to assess TPI in calves, this technique can be used to understand the effect of time on the dynamics of saliva Ig concentrations in newborn calves and contribute to validating the use of saliva to assess TPI with other on-farm tools, such as Brix refractometer [15] or calf-side tests [50].

## 5. Conclusions

Saliva Ig concentrations in newborn calves are affected by colostrum intake and composition. Furthermore, saliva IgG and IgA concentrations are related to their serum counterparts, thus indicating their potential to predict serum IgG and IgA concentrations after colostrum intake. The small sample size of the study is a limitation and, therefore, further research with more animals is recommended to provide a robust conclusion about the suitability of saliva as a sample for assessing TPI in newborn dairy calves.

## Figures and Tables

**Table 1:** Descriptive statistics of colostrum nutritional and immunological composition.

Variable	Mean	SD	Median	IQR
Dry matter, %	24.0	3.87	24.2	6.4
Protein, %	13.6	1.75	14.5	2.8
Fat, %	4.8	2.92	5.1	4.9
IgG, mg/mL	44.7	16.56	45.9	24.3
IgA, mg/mL	2.5	1.70	2.4	2.0
IgM, mg/mL	2.3	1.86	2.2	1.7
SCC, cells^6^/mL	3.1	2.93	2.5	4.5

Abbreviations: SD, standard deviation; IQR, interquartile range; SCC, somatic cell count.

**Table 2:** Final multiple regression models evaluating the association between colostrum composition (protein, fat, DM, SCC, IgG, IgA, and IgM), colostrum intake (C.INT), and calf age and saliva and serum Ig (i.e., IgG, IgA, and IgM) and TP on the first day of life (d 1) ^1^.

Outcome Variable	Explanatory Variables	Coefficient	SE	*p*-Value	R^2^
Saliva variables					
IgG	Intercept	−565.14	226.81	0.028	0.482
	Colostrum SCC ^2^	44.74	23.39	0.080	
	C.INT	112.19	34.20	0.007	
IgM	Intercept	−46.50	12.55	0.005	0.73
	Colostrum IgA	1.78	0.71	0.034	
	Colostrum IgG	−0.15	0.05	0.016	
	Colostrum SCC ^2^	3.38	1.12	0.015	
	Colostrum protein	1.06	0.56	0.092	
	C.INT	4.86	1.56	0.012	
TP	Intercept	−0.22	0.13	0.117	0.407
	Colostrum IgM	0.02	0.01	0.046	
	C.INT	0.08	0.03	0.026	
Serum variables					
IgG	Intercept	−25.90	13.50	0.079	0.486
	Colostrum protein	1.41	0.72	0.072	
	C.INT	7.10	2.74	0.024	
IgA	Intercept	−6984.32	2540.95	<0.001	0.914
	Colostrum IgA	1085.36	170.66	<0.001	
	Colostrum IgM	−54.19	107.57	0.046	
	Colostrum IgG	−26.49	10.65	0.038	
	Colostrum protein	455.13	125.98	0.007	
	C.INT	877.89	335.97	0.031	
	Age	−5.68	3.79	0.172	
IgM	Intercept	−1003.06	543.98	0.095	0.657
	Colostrum IgM	55.06	41.79	0.217	
	Colostrum IgA	117.42	56.55	0.065	
	Colostrum SCC ^2^	125.78	90.67	0.196	
	Age	3.27	1.22	0.024	
TP	Intercept	0.006	1.10	0.996	0.889
	Colostrum IgM	−0.188	0.06	0.011	
	Colostrum protein	0.303	0.05	<0.001	
	C.INT	0.784	0.207	0.003	

^1^ Final coefficients, standard errors (SEs), and significance levels (*p*-values) of the predictors retained in the backward stepwise selection models. ^2^ Expressed in log_10_ cells/mL. Abbreviations: C.INT, colostrum intake; SCC, somatic cell count; TP, total protein; Age, age in hours at first colostrum intake.

**Table 3:** Predictive models of serum IgG concentration in newborn calves based on saliva IgG concentration: comparison between models including and excluding time (days 0, 1, 2, and 7) ^#^.

Terms	Coefficient	SE	*p*-Value
Model with the effect of time ^§^			
Intercept	4.72	1.29	0.0005
Saliva IgG	100.93	15.76	<0.0001
Time	1.73	0.40	<0.0001
Saliva IgG × Time	−19.67	6.67	0.0016
Model without the effect of time ^&^			
Intercept	12.25	0.74	<0.0001
Saliva IgG	36.58	6.73	<0.0001
Saliva IgG^2^	−20.61	6.73	0.0030

^#^ Coefficients, standard errors (SEs), and *p*-values for linear mixed-effects models predicting serum IgG from saliva IgG. The model with time includes saliva IgG, time (as a continuous covariate), and their interaction. The model without time includes a quadratic term for saliva IgG. Random intercepts for calf were included in the model with time. ^§^ R^2^ = 0.40. ^&^ R^2^ = 0.33.

**Table 4:** Predictive models of serum IgA concentration in newborn calves based on saliva IgA concentration: models including and excluding time (days 0, 1, 2, and 7) ^1^.

Terms	Coefficient	SE	*p*-Value
Model with the effect of time ^2^			
Intercept	1.08	0.18	<0.001
Saliva IgA	0.005	0.002	0.016
Time (d 1)	5.37	0.24	<0.001
Time (d 2)	4.72	0.22	<0.001
Time (d 7)	2.47	0.22	<0.001
Model without the effect of time ^3^			
Intercept	3.53	0.38	<0.001
Saliva IgA	0.02	0.006	<0.001

^1^ Coefficients, standard errors (SEs), and *p*-values for linear models predicting serum IgA from saliva IgA. The model with time includes time as a categorical variable (considering d 0 as the reference category), and the response variable was transformed to the 0.25 power. The model without time includes saliva IgA as the sole predictor. No random effects were used in either model. ^2^ R^2^ = 0.91. ^3^ R^2^ = 0.13.

## Data Availability

Data is available upon direct request to the corresponding authors.
